# The Possible Role of *Bifidobacterium longum* BB536 and *Lactobacillus rhamnosus* HN001 on Locomotor Activity and Oxidative Stress in a Rotenone-Induced Zebrafish Model of Parkinson's Disease

**DOI:** 10.1155/2021/9629102

**Published:** 2021-10-14

**Authors:** Ovidiu-Dumitru Ilie, Emanuela Paduraru, Madalina-Andreea Robea, Ioana-Miruna Balmus, Roxana Jijie, Mircea Nicoara, Alin Ciobica, Ilinca-Bianca Nita, Romeo Dobrin, Bogdan Doroftei

**Affiliations:** ^1^Department of Biology, Faculty of Biology, “Alexandru Ioan Cuza” University, Carol I Avenue, No 20A, 700505 Iasi, Romania; ^2^Faculty of Geography and Geology, “Alexandru Ioan Cuza” University, Carol I Avenue, No 20A, 700505 Iasi, Romania; ^3^Department of Exact and Natural Sciences, Institute of Interdisciplinary Research, “Alexandru Ioan Cuza” University, Carol I Avenue, No 11, 700506 Iasi, Romania; ^4^Faculty of Medicine, University of Medicine and Pharmacy “Grigore T. Popa”, University Street, No 16, 700115 Iasi, Romania; ^5^Department of Psychiatry, Faculty of Medicine, University of Medicine and Pharmacy “Grigore T. Popa”, University Street, No 16, 700115 Iasi, Romania

## Abstract

**Background:**

As every organ within the body, the brain is also extremely susceptible to a plethora of noxious agents that change its chemistry. One component frequently found in current products against harmful species to crops is rotenone whose effect under prolonged exposure has been demonstrated to cause neurodegenerative disorders such as Parkinson's disease. The latest reports have indeed revealed that rotenone promotes Parkinson's in humans, but studies aiming to show congruent effects in zebrafish (*Danio rerio*) are lacking. *Material and Methods*. In this context, the aim of the present study was to demonstrate how chronic administration of rotenone for 3 weeks impairs the locomotor activity and sociability and induces oxidative stress in zebrafish.

**Results:**

There were no statistically significant differences following the analysis of their social interaction and locomotor tests (*p* > 0.05). However, several exceptions have been noted in the control, rotenone, and probiotics groups when we compared their locomotor activity during the pretreatment and treatment interval (*p* < 0.05). We further assessed the role of rotenone in disturbing the detoxifying system as represented by three enzymes known as superoxide dismutase (SOD), glutathione peroxidase (GPx), and malondialdehyde (MDA). Despite the fact that there were no statistically significant changes within SOD and GPx levels between the control group and rotenone, probiotics, and rotenone + probiotics (*p* > 0.05), relevant changes have been observed between the analyzed groups (*p* < 0.05 and *p* < 0.005, respectively). On the other hand, significant differences (*p* < 0.05) have been observed for MDA when we analyzed the data between the control group and the other three groups.

**Conclusions:**

Our results suggest that rotenone can be successfully used to trigger Parkinson's disease-related symptomatology in zebrafish.

## 1. Introduction

Parkinson's disease (PD) is the second most common neurodegenerative disorder after Alzheimer's disease (AD) and is expected to advance by 2050. According to the latest published statistics, the incidence and prevalence fluctuate around 4.5-21, respectively, 18-328 cases at 100,000 individuals per year (~10 million worldwide). Although of onset is in the range of the fourth and seventh decades of life, the existing evidence refutes this hypothesis [1].

Mechanistically speaking, PD is characterized by a constant loss of dopaminergic and cholinergic neurons from the substantia nigra pars compacta (SNpc) and posterior motor nucleus of the vagus. Furthermore, it is characterized by the continuous storage and aggregation of the *α*-synuclein within the central nervous system (CNS) [2].

Despite best efforts, the etiology of PD remains obscure, recent studies revealing that PD actually possesses a multifactorial substrate, reflected by major phenotypic changes. Specifically, the clinical signs exhibited by patients are grouped under the umbrella of the Parkinsonism spectrum and include essential tremor, stiffness, bradykinesia, and impaired postural balance [2].

Regarding its origin, PD has been shown to be caused by exposure to exogenous stressors (pesticides, heavy metals, or illicit substances) [3], either by abnormal gene expression, with or without family aggregation. By default, these determinants may gradually induce autosomal dominant PD or autosomal recessive juvenile PD [4]. However, people diagnosed and treated with antipsychotics are prone to significant side effects, a phenomenon known as drug-induced Parkinson's syndrome (PID) [5].

Cumulatively, it can be argued that PD is gradually triggered by various factors, but there are also situations in which the cause is idiopathic. Here was another long-time discussion due to the clinical heterogeneity and overlap between PD dementia, Lewy body dementia, and other types, which is why the nomenclature has been constantly reevaluated [6].

One common and usually underexplored feature of PD patients, yet crucial, is the role of anxiety [7] and depression [8] as promoters and/or pointers of PD. Analogous for healthy patients exposed to prolonged stress states as a risk group. The associated changes of the brain's chemistry have been extensively discussed from both perspectives [9, 10], and especially the petulant and abnormal oxidative stress (OS) that disrupts the physiological status of the redox potential [11]. The missing link of this puzzle could be the gut microbiota due to its ability to shape humans behavior and development, regardless of age, sex, and health status [12].

The most powerful vehicle used in clinical practice to diminish PD-related symptomatology and the associated comorbidities causative or not are probiotics [13]. These are live microorganisms known to have a helpful role in improving digestive function [14, 15]. Their implication in PD was often suggested in the last years [13, 16, 17]. Along this, there are not too many studies to sustain their importance in the PD diet but, until now, the evidence strongly supports probiotics [18]. It has been demonstrated in three distinct randomized controlled trials that probiotics have the capacity to ameliorate all related-gastrointestinal deficiencies (constipation) in patients compared with the placebo. More precisely, the authors administered between 1 and 3 month strains of *Streptococcus*, *Enterococcus*, and especially *Bifidobacterium* and *Lactobacillus* and observed an improvement in life's quality, changes in fecal calprotectin, and less bowel deficiencies. However, the results following Unified Parkinson's Disease Rating Scale remained low despite the therapy [18–20].

Various animal models are being used in modeling PD disorder in order to observe the mechanisms or even possible find new therapeutic interventions in ameliorating and/or treating it. The successful usage of *Danio rerio* as a model for neurologic disorders is due to its main advantages such as physiological homology to human individuals, short time of replication, and low cost of breeding as compared to other experimental models, and it is also suitable for drug development studies [21]. Its implication in PD modeling was described and published in many reports over the years [22–27].

The pesticide rotenone was often used for inducing PD symptoms in rodents but also there are several studies which assessed rotenone's impact on zebrafish [28–31]. Rotenone was for a long time utilized in several commercialized insecticides, pesticides, or piscicides [24]. Beside behavioral aspects, rotenone is able to intervene in the OS balance by influencing enzymes level responsible with antioxidant role [32].

Having as support all these data, the aim of this study was to determine if probiotics brought a beneficial role in reducing the oxidative status and reestablishment potential of the motor impairment in a zebrafish PD model.

## 2. Material and Methods

### 2.1. Animal Husbandry

A total of sixty adults (6-8 months old) wild-type (WT) zebrafish (*Danio rerio*) were purchased from a local breeder. The fish were accommodated three weeks to laboratory conditions and kept in a 90 L recirculating dechlorinated water aquarium. Both aquariums (housing and experimental) respected the standard parameters as 26 ± 2°C temperature, pH 7.5, with a 14 h light/10 h night cycle [33]. Water was changed daily in the experimental tanks. The adults were fed twice per day with TetraMin Flakes.

### 2.2. Ethical Approval

This experiment was performed by respecting the EU Commission Recommendation (2007), Directive 2010/63/EU of the European Parliament, and the Council of 22 September 2010 regarding protection, accommodation, and care of animals for scientific/experimental purposes [34, 35]. The current protocol was approved by the Ethical Committee of the Faculty of Biology, “Alexandru Ioan Cuza” University of Iasi with the registration number 11/25.05.2021.

### 2.3. Chemicals

#### 2.3.1. Rotenone

Rotenone (C_23_H_22_O_6_) was purchased from Toronto Research Chemicals, North York, Canada (Cat# R700580), under a white powder that has been dissolved in distilled water until we reached a 2 *μ*g L^−1^ concentration that was further administered daily for 21 days [30, 36].

#### 2.3.2. Probiotics

Zircombi (ALFASIGMAS.p.A.) is a dedicated food supplement for people, having the appearance of a white powder that was purchased from a local pharmacy. It contains two strains—*Bifidobacterium longum* BB536-4 × 10^9^ CFU (150 mg) and *Lactobacillus rhamnosus* HN001-1 × 10^9^ CFU (25 mg) and Vit B_6_-1.4 mg. We dissolved this mixture in ¼ water using a 100 ml rated balloon being administered half an hour before routine feeding in rearing water to ensure the ingestion as indicated by Valcarce et al. and obtaining as follows: 3 mg L^−1^ BB536, 0.5 mg L^−1^ HN001, and 0.02 mg L^−1^ vit. B_6_ [37].

### 2.4. Design Protocol

After the accommodation period was over, they were subsequently divided into four equal groups (*n* = 15) and placed in small 10 L tanks for another two days in order to adapt to the new environment. Afterward, we assessed their swimming performance and sociability to determine whether or not are differences between pretreatment and treatment period. The groups were as follows: group 1 (control), group 2 (probiotics), group 3 (rotenone), and group 4 (rotenone + probiotics). The substances were daily renewed after water changing. The entire study lasted 21 days, and at the end of the experimental period, fishes were killed in water with ice at a temperature under 5°C [38].

### 2.5. Behavioral Assessment

**Figure 1:**
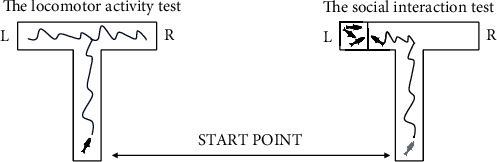
The experimental designs of zebrafish behavioral testing adapted to T-maze.

The social interaction test was performed daily to evaluate the changes induced by rotenone and the possible behavior benefic role of probiotics. For this, we used a modified cross maze labyrinth closed by a slit and transformed into a T maze (10 h × 50 l × 50 w cm) filled with system water (5 cm) [39, 40]. The social stimulus was placed in the left arm of the T maze (Figure 1). The locomotor activity test was performed in the same maze. Once introduced, each fish was let to acclimate to the new conditions for half a minute. Movement was registered with a professional camera placed above the experimental chamber over a period of 240 seconds. Parameters were analyzed using EthoVision XT 11.5 software (Noldus Information Technology, Wageningen, The Netherlands). We focused on 3 specific parameters such as the total distance swam (cm), velocity (cm s^−1^), and active status (s) for locomotor activity test and the time spent in the left arm (s) for social interaction test.

### 2.6. Oxidative Stress Measurement and Sample Preparation

Superoxide dismutase determination kit (SOD, 19160-1KT-F), Glutathione Peroxidase Cellular Activity Assay Kit (GPx, CGP1-1KT), and Protein Quantification Kit-Rapid (51254-KT) were purchased from Merck, Germany. All the analyses were performed according to the manufacturer's instructions. Malondialdehyde (MDA) levels were assessed by thiobarbituric acid-reactive assay following a preestablished work protocol [41].

At the end of chronic exposure period, each fish was individually well homogenized in a 10 volume of ice-cold saline (0.90% NaCl). Each sample was centrifuged at 5500 rpm for 10 min in accordance with the already established protocols by Jin et al. [42] and Ni et al. [43]. The supernatant obtained was aliquoted into 2 ml Eppendorf tubes for the subsequent determination of oxidative biomarkers levels. A spectrophotometer at distinct wavelengths (Specord 210 Plus producer Analytik Jena, Germany) was used to assess each enzyme activity.

### 2.7. Statistical Analysis

Through the Shapiro-Wilk test, we first analyzed the normality and distribution of the data, Microsoft Excel 2010 being used for editing, sorting, and coding of the raw data. The data was then exported into OriginPro v.9.3 (2016) software (OriginLab Corporation, Northampton, MA, USA). Thereafter, we used one-way ANOVA and Tukey HSD test to verify and certify whether or not there are significant different variances among investigated parameters from the start until endpoint [39, 44]. For the abovementioned behavioral parameters, data are presented as average ± SEM. For OS, we calculated the variance between the control and the experimental groups performing multiple comparisons; data expressed as average ± SEM. *p* < 0.05 was regarded as statistically significant.

## 3. Results

### 3.1. The Impact of Rotenone and Probiotics on Zebrafish Swimming Performance

According to our swimming performance, parameters as the total distance swam and the average velocity showed no pronounced effects of rotenone or probiotics administration after 21 days. Regarding the total distance swam, all the experimental groups recorded several changes between pretreatment and treatment period. The control group showed several picks of activity during experimental period compared to the pretreatment days as: D_10 (1240.03 ± 169.9 cm, *p* = 0.01 Tukey, ANOVA), D_13 (1209.58 ± 71.7 cm, *p* = 0.02 Tukey, ANOVA), and D_15 (1220.13 ± 104.2 cm, *p* = 0.02 Tukey, ANOVA) vs. 620.7 ± 127.8 cm. The second group exposed to rotenone did not present significant changes during the treatment excepting D_6 (1369.2 ± 255.8 cm, *p* = 0.008 Tukey, ANOVA) and D_14 (1642.9 ± 165.1 cm, *p* = 0.002 Tukey, ANOVA).

**Figure 2:**
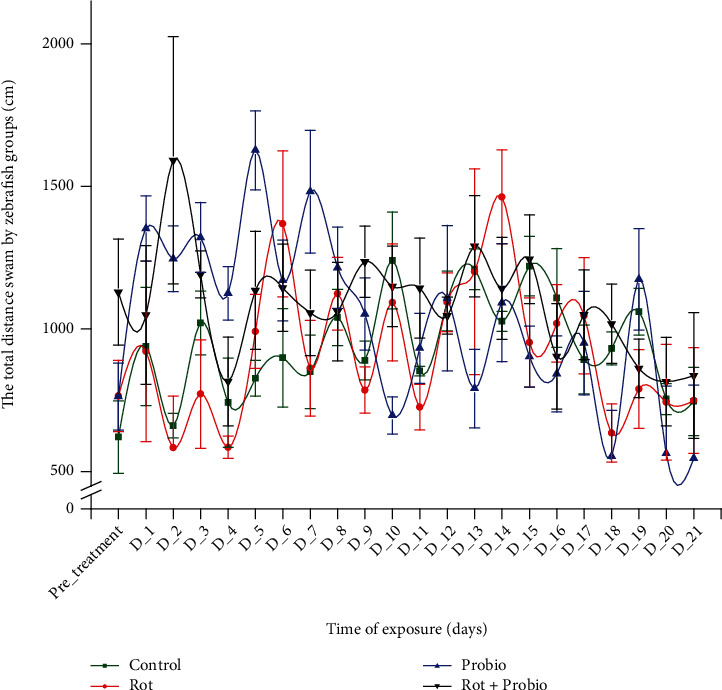
The total distance swam by experimental groups during locomotor activity test (*n* = 15). Green: control group, red: rotenone (2 *μ*g L^−1^), blue: probiotics (3 mg L^−1^ BB536, 0.5 mg L^−1^ HN001, and 0.02 mg L^−1^ vit. B_6_), black: rotenone (2 *μ*g L^−1^) and probiotics (3 mg L^−1^ BB536, 0.5 mg L^−1^ HN001, and 0.02 mg L^−1^ vit. B_6_). The groups mean was compared to pretreatment days mean, and the results were represented as average ± SEM.

The third group treated with probiotics showed an increase of distance swam in the first days of the treatment with a highest value for the D_5 (1626.4 ± 138.2 cm, *p* = 0.006 Tukey, ANOVA). After 1 week of administration, the trend was composed from ups and downs as it can be seen in Figure 2. When rotenone and probiotics were administrated together, the activity of this parameter did not record important modifications (*p* > 0.05 ANOVA).

**Figure 3:**
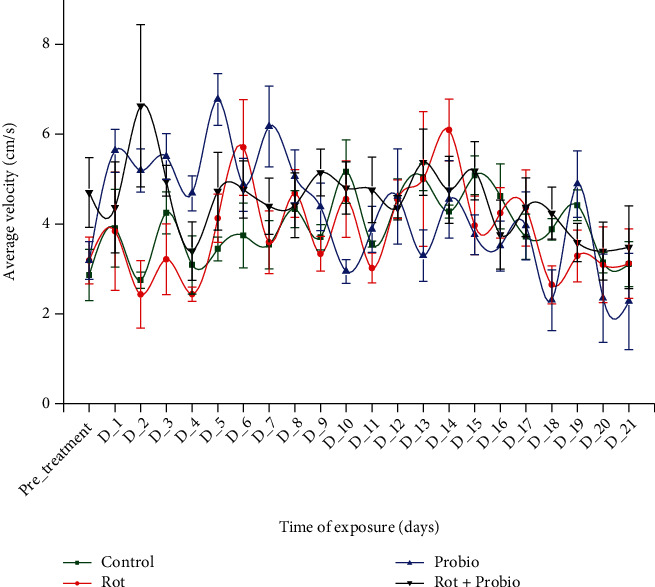
The average velocity of the experimental groups recorded during locomotor activity test (*n* = 15). Green: control group, red: rotenone (2 *μ*g L^−1^), blue: probiotics (3 mg L^−1^ BB536, 0.5 mg L^−1^ HN001, and 0.02 mg L^−1^ vit. B_6_), black: rotenone (2 *μ*g L^−1^) and probiotics (3 mg L^−1^ BB536, 0.5 mg L^−1^ HN001, and 0.02 mg L^−1^ vit. B_6_). The group mean was compared to pretreatment days mean, and the results were represented as average ± SEM.

No significant changes were observed for control, rotenone, and mixture group regarding the velocity parameter (*p* > 0.05 ANOVA). The trend was similar for the probiotic group excepting the short boost of hyperactivity in D_5 (6.77 ± 0.57 cm/s, *p* = 0.005 Tukey, ANOVA) compared to the pretreatment: 3.18 ± 0.42 cm/s (Figure 3).

**Figure 4:**
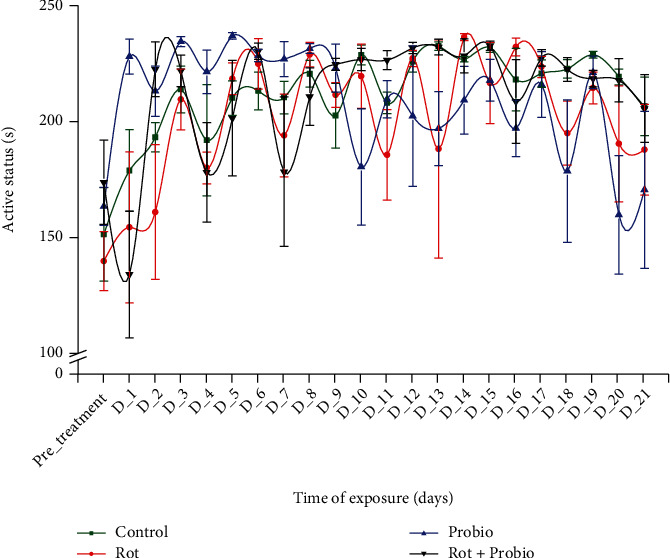
The active status of the experimental groups recorded during locomotor activity test (*n* = 15). Green: control group, red: rotenone (2 *μ*g L^−1^), blue: probiotics (3 mg L^−1^ BB536, 0.5 mg L^−1^ HN001, and 0.02 mg L^−1^ vit. B_6_), black: rotenone (2 *μ*g L^−1^) and probiotics (3 mg L^−1^ BB536, 0.5 mg L^−1^ HN001, and 0.02 mg L^−1^ vit. B_6_). The groups mean was compared to pretreatment days mean, and the results were represented as average ± SEM.

We also determined the active status which measures the time spent by fish being active during the session. In the pretreatment days, fish from the control group showed a decrease in time moving, but its activity during the whole experimental period was following a constant trend. Fish exposed to rotenone recorded increases in time spent moving as D_6 (224.8 ± 10.8 s, *p* = 0.03 Tukey, ANOVA), D_8 (228.8 ± 5.22 s, *p* = 0.02 Tukey, ANOVA), D_12 (227.2 ± 3.52 s, *p* = 0.02 Tukey, ANOVA), D_14 (236.3 ± 1.50 s, *p* = 0.007 Tukey, ANOVA), D_16 (232.1 ± 3.78 s, *p* = 0.01 Tukey, ANOVA), and D_17 (223.5 ± 4.55 s, *p* = 0.04 Tukey, ANOVA) in comparison to 139.8 ± 12.7 s of pretreatment time. Regarding the activity for the third group treated with probiotics, its activity was similar with those observed for the abovementioned parameters and increases in the first part of the administration and then ups and downs. However, there was no statistically significant difference for the mixture group (*p* > 0.05ANOVA) (Figure 4).

### 3.2. No Effect of Rotenone and Probiotic Administration on Zebrafish Sociability

**Figure 5:**
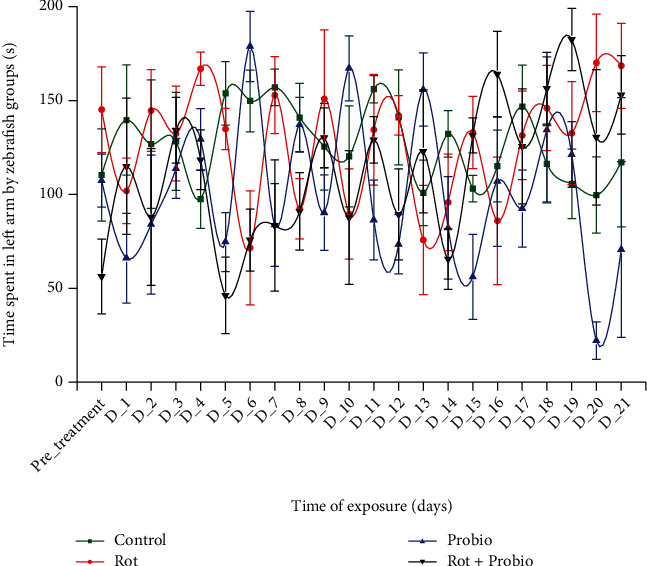
The time spent in the left arm by the experimental groups recorded during social interaction test (*n* = 15). Green: control group, red: rotenone (2 *μ*g L^−1^), blue: probiotics (3 mg L^−1^ BB536, 0.5 mg L^−1^ HN001, and 0.02 mg L^−1^ vit. B_6_), black: rotenone (2 *μ*g L^−1^) and probiotics (3 mg L^−1^ BB536, 0.5 mg L^−1^ HN001, and 0.02 mg L^−1^ vit. B_6_). The groups mean was compared to pretreatment days mean, and the results were represented as average ± SEM.

No significant changes were observed for the control and rotenone group (*p* > 0.05 ANOVA). The single administration of rotenone or probiotics did not have any effect on zebrafish sociability after 21 days of exposure as it can be seen in Figure 5. Moreover, a similar effect was also observed for the last group excepting D_19 when time spent next to the group increased considerably compared to pretreatment period (182.5 ± 16.5 s vs. 56.2 ± 19.8 s, *p* = 0.01 Tukey, ANOVA).

### 3.3. Oxidative Stress

**Figure 6:**
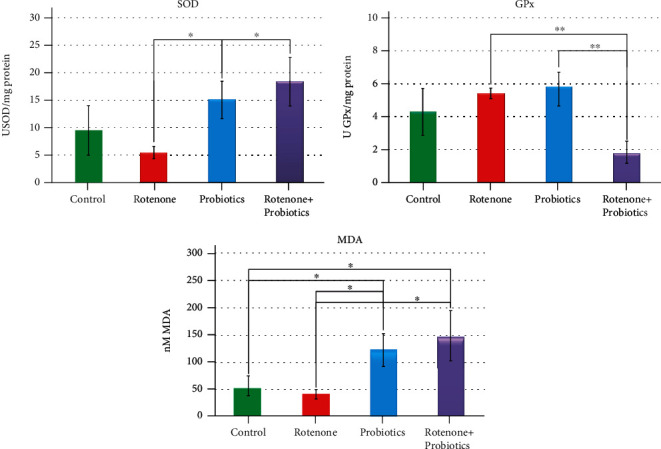
The activity of superoxide dismutase (SOD), glutathione peroxidase (GPx), and malondialdehyde (MDA) levels in zebrafish chronically exposed to rotenone and probiotics. Data were expressed as average ± SEM (^∗^*p* < 0.05; ^∗∗^*p* < 0.005).

While analyzing the data regarding OS after 21 days of rotenone administration in zebrafish individuals, we noted several changes (Figure 6). For SOD, there was no statistically significant difference between the control group and the other three (*p* > 0.05) but rather when we compared the rotenone group with probiotics (*p* = 0.014) and probiotics + rotenone (*p* = 0.011). SOD level dropped in the rotenone-exposed group compared to the control group. Instead, the probiotics group showed an increase in SOD activity, result which was expected to obtain knowing the fact that probiotics can enhance SOD expression in living cells [45] and the differential response of each body depending on the organ investigated [46]. Meanwhile, the last group exposed to rotenone and probiotics had also an increase in SOD level but still with no considerable change compared to control.

In the case of GPx, analogous results were obtained since there were no statistically significant differences between the control group and the rest of the groups investigated (*p* > 0.05). On the other hand, there was a significant change of GPx level when compared rotenone group with probiotics (*p* = 0.001) and rotenone + probiotics (*p* = 0.004) groups. It is already known that OS is often reported as a risk factor in PD development [47–49]. SOD and GPx are considered to be the first line defense against free radicals [50] and a diet supplementation with *Lactobacillus fermentum* enhance the antioxidant system as reported by Wang et al. [51]. A similar pattern has been also observed for GPx despite the fact that no significant difference, whereas the level of SOD is compensatory increased. Based on these considerations, there is certainty of the existence of OS. The possible explanation is that Bifidobacterium longum BB536 and Lactobacillus rhamnosus HN001 modulate anxiety state in Danio rerio but at the same time promote a hyperlocomotor activity [52], hence, these abnormal levels of OS biomarkers.

Furthermore, the main marker of lipidic peroxidation, MDA, presented significant high levels (*p* < 0.05) in the control group as compared with probiotics (*p* = 0.033) and in contrast with rotenone and probiotics (*p* = 0.032). Also, there were significant changes in terms of MDA levels between the rotenone group and probiotics (*p* = 0.009) and rotenone + probiotics (*p* = 0.01) groups, respectively. Congruent with other studies, it appears that MDA is significantly increased after 30 days of 2 mg L^−1^ rotenone administration [46], *Bifidobacterium longum* BB536 being a reliable bacteria attributed to the reestablishment of the antioxidant system [53].

## 4. Discussion

A variety of reports have highlighted that fish, among other aquatic organisms, are deeply sensitive to pollutants and also to natural compounds used as pesticide. In our study, chronic administration of rotenone was performed in zebrafish adults, and its effect on behavior and OS was measured. We observed that administration of 2 *μ*g L^−1^ rotenone had impacted the total distance travelled during test session presenting ups and downs compared to the pretreatment time. For example, Khotimah et al. [54] reported a decreased motility after 2 weeks of 5 *μ*g/L rotenone administration. As already reported by Wang et al. [30] and our team, there were no significant differences in terms of locomotor impairment, thus, further highlighting the fact that PD triggering in *Danio rerio* is dependent on dosage and time of administration. These changes in locomotor activity can be a consequence of mitochondrial dysfunction which is responsible with ATP production or/and overproduction of free radicals. It was suggested that these abovementioned can lead to dopamine deficiency and apoptosis by the study of Khotimah and his team in 2015 after chronic treatment of zebrafish with 5 *μ*g L^−1^ rotenone for 28 days [54]. One year later Khatri and Juvekar also evaluated the influence of 5 *μ*g L^−1^ rotenone on swimming behavior after 14 days of treatment [55].

When rotenone and probiotics were given together, this parameter did not show any significant changes. This could be an effect triggered by probiotic presence in the medium based on the third group results exposed only to probiotics where their activity was particularly increased in the first week of the treatment than the normal behavior recorded in the pretreatment.

It is interesting to observe that active status was different in the case of the second and forth group which received rotenone, respectively, rotenone and probiotics in the first day of treatment. Rotenone administration led to a hazardous zebrafish activity for all the period in comparison to the rotenone and probiotics group whose activity was adjusted after only 1 week of probiotic treatment.

To determine if rotenone has certain effects on zebrafish sociability the social interaction test was performed daily. According to our data, no significant impact on sociability was registered, fish manifesting contradictory behavior day after day. Even probiotic administration did not induce important advances in zebrafish social domain; it just sustains the normal activity. It was showed that the administration of probiotics together with oxytocin could have a positive influence on social domain in an ASD mouse model [56].

OS is a common risk factor often reported in PD pathogenesis [48, 57]. Described as imbalance between antioxidants and oxidants, OS generates serious cellular damage which causes, mainly, neuronal degeneration [58]. In our study, we observed several changes in the antioxidant enzymes activity and an increased level of lipidic peroxidation. Treatment with 2 *μ*g L^−1^ rotenone for 21 days leads to a decrease in SOD level which was in agreement with Khan's study observations made on PD patients regarding OS markers [49]. On the other hand, SOD production was encouraged by probiotics. For example, diet supplementation with *Lactobacillus fermentum* could increase the level of SOD and GPx in pigs [51].

Exposing adult zebrafish to 2 mg L^−1^ rotenone for 30 days showed significant activity of MDA and decreased SOD level both intestines and brain for glutathione-S-transferase (GST) and catalase (CAT) level [46]. Neurodegeneration and OS have also been recorded after experimental exposure of human liver HepG2 cells to a range between 12.5 and 250 *μ*M rotenone for 24 hours [59]. If rotenone favors OS, probiotic presence can stimulate the antioxidant system as it was demonstrated in our study by high levels of SOD and GPX.

Due to potentially antioxidant role, probiotics started to receive more attention, and since OS was linked to altered gut microbiota. The gut-brain axis is an important interaction which has been reported to be disrupted in PD individuals [60–62].

For example, Bhattaraiet al. and Dodiya and coauthors [63, 64] administered 10 mg/kg/day of rotenone for six and twelve weeks, respectively, and brought several solid arguments in this context. The administration for a period of 6 weeks led to a loss of tyrosine hydroxylase (TH) neurons in both conventionally raised mice and germ-free. In parallel, it resulted in a decrease in motor strength and coordination. The researchers concluded that chronic rotenone intake did not cause dysbiosis, rather a shift among bacterial load and increased in the first month and permeability in conventionally raised mice. On the other hand, it promoted elevated urinary cortisol, intestinal hyperpermeability, and a diminished abundance of lactic bacteria (*Lactobacillus*) in restraint stress mice by comparison with the control during the first month and a half. Moreover, rotenone alone without restraint stress disrupted the colonic expression of the tight junction protein ZO-1 and increased the protein level of *α*-syn in the colon, N-tyrosine as a marker of OS, and myenteric plexus enteric glial cell-glial fibrillary acidic protein expression by comparing with the control group. In the restraint stress rotenone-induced was observed analogous changes as in the rotenone alone group with several exceptions; intestinal hyperpermeability, an abnormal expression of Occludin and Claudin1, and increased ratio of fecal *Akkermania* and endotoxemia. Nevertheless, an intraperitoneal injection of 2.75 mg/kg rotenone, 5 days per week for 1 month promoted GI dysfunctionalities like diarrhea and delayed gastric emptying. The subsequent microbiota analyses revealed alterations along the small intestine and colon, whereas histological examination indicates a mucosal thickening and goblet cell hyperplasia in the colon [65].

Nonetheless, *Bifidobacterium longum* BB536 and *Lactobacillus rhamnosus* HN001 are two probiotic strains that have been previously administered also in patients suffering from irritable bowel syndrome (IBS). Specifically, there was a statistically significant decrease (*p* < 0.0001) within two parameters regarding “abdominal pain” and “bloating” and overall severity of disease between IBS patients and placebo. The beneficial effects were more detectable in IBS-diarrhea patients as indicated by the Bristol scale (*p* < 0.00001) in contrast with placebo (*p* = 0.04), while in IBS-constipation, there was only a slight improvement (*p* = 0.06) [66]. Similar findings were made by another team in which they enrolled lactose intolerance patients vs. placebo. This time, the physical symptoms panel as indicated by “bloating” (*p* = 0.028) and “constipation” (*p* = 0.045) were ameliorated in lactose intolerance patients as compared to placebo.

Another preliminary study revealed an increased load of both these species in pre- and postprandial individuals that persisted even after one month following the end of oral intake. At the phyla level, there was observed a significant decrease of *Firmicutes*, *Proteobacteria* as compared with the sample collected prior to the actual procedure. At the species level, the authors noted an increased abundance of several microorganisms including *Blautia producta/wexlerae*, *Haemophilus ducrey*, *Akkermansia muciniphila*, *Roseburia faecis*, and *Ruminococcus gnavus* during follow-up, followed by a reduction of *Holdemania filiformis*, *Escherichia vulneris*, *Gemmiger formicilis*, and *Streptococcus sinensis* [67].

This mixture has been also successfully administered in rat models. According to the results of Alsheraji et al., [53] *Bifidobacterium longum* BB536 reduced total cholesterol, very-low, low and high-density lipoprotein cholesterol, and atherosclerotic index amounts, concomitantly with that of liver-lipid deposition and adipocyte size. In the same study, they demonstrated that the plasma MDA level significantly decreased after the administration of *Bifidobacterium longum* BB536.

*Bifidobacterium longum* BB536 and *Lactobacillus rhamnosus* HN001, in combination and/or individually, possess the ability to inhibit the activity of the major Gram-negative strains, even opportunistic pathogenic entities such as *Candida* [68, 69]. Different teams of researchers have also revealed in several other occasions that rotenone causes a dysfunction of the rat's and mice's gastrointestinal microbiota as well. Precisely, there was observed a decrease of *Bifidobacterium* genus, as well as in *Firmicutes* and *Bacteroides* phyla [70, 71].

## 5. Conclusions

It can be concluded based on our results that rotenone is a potent toxic agent that can be successfully used to trigger PD-related symptomatology in *Danio rerio.* Furthermore, it can offer a pathway to improve our knowledge regarding the etiology of PD, and these observations can be in the future further extrapolated to human individuals. Even though *Bifidobacterium longum* BB536 and *Lactobacillus rhamnosus* HN001 did not improve the locomotor activity, nor diminish oxidative status in zebrafish but rather induced a hyper-activity, our study offered a new perspective in contrast with the existing information.

## Data Availability

The datasets used and analyzed during the current study are available from the corresponding author on reasonable request.
